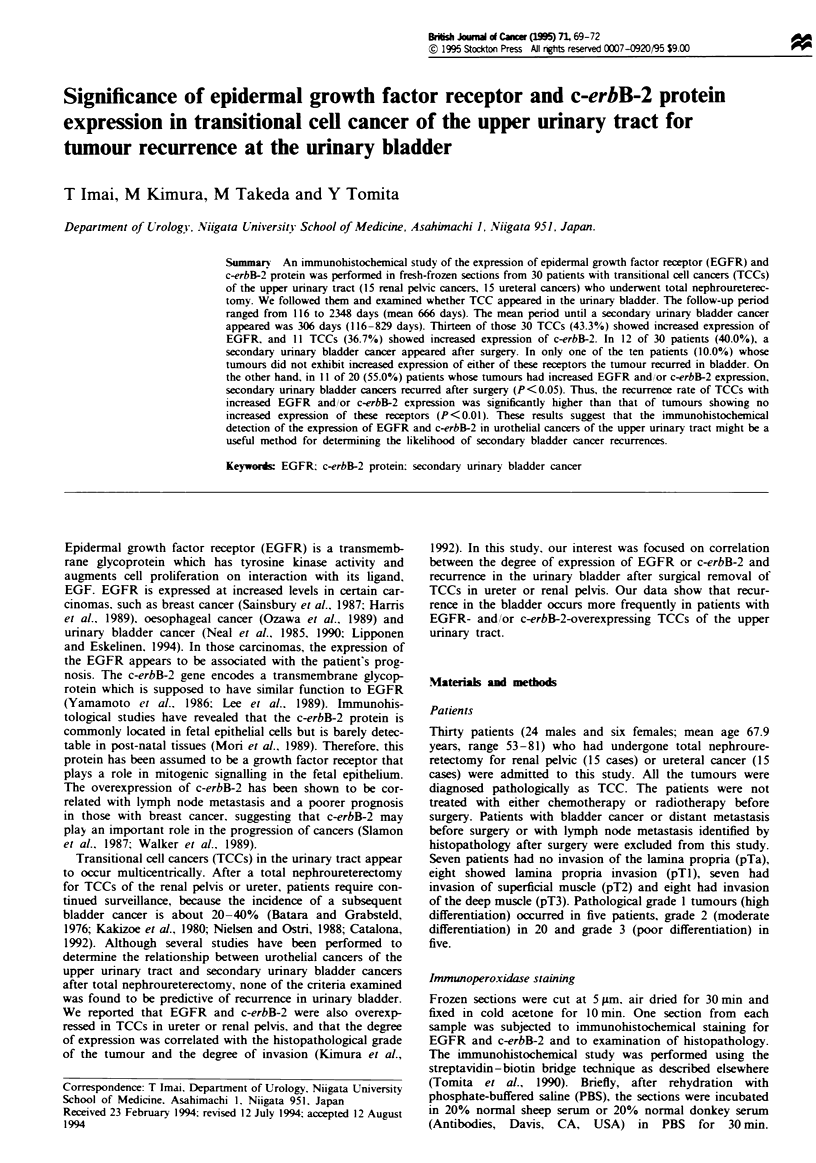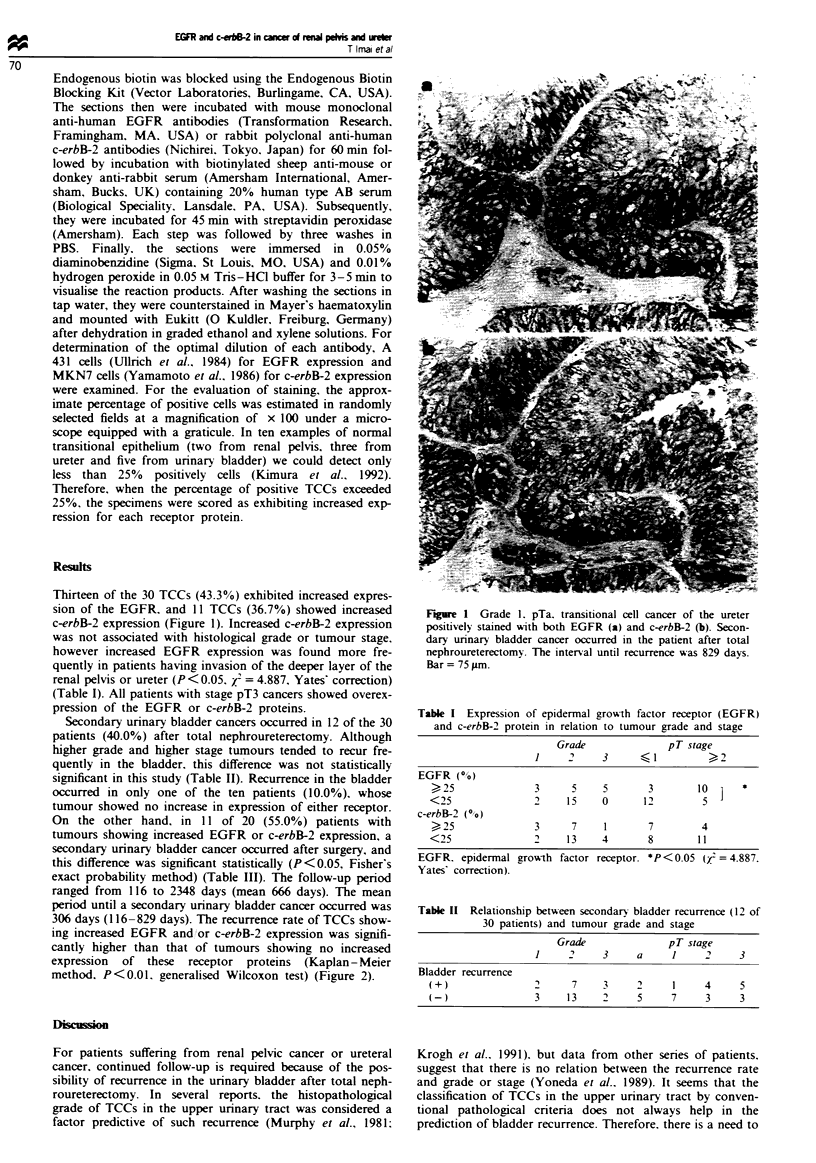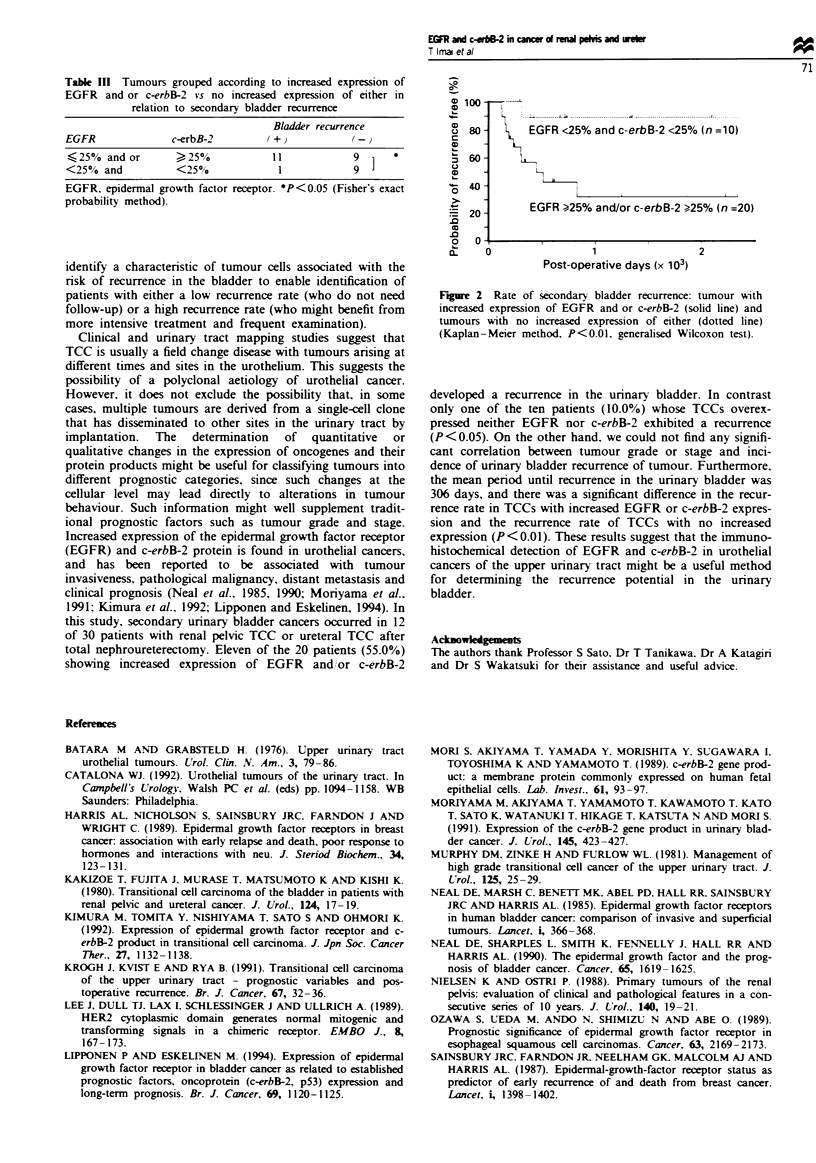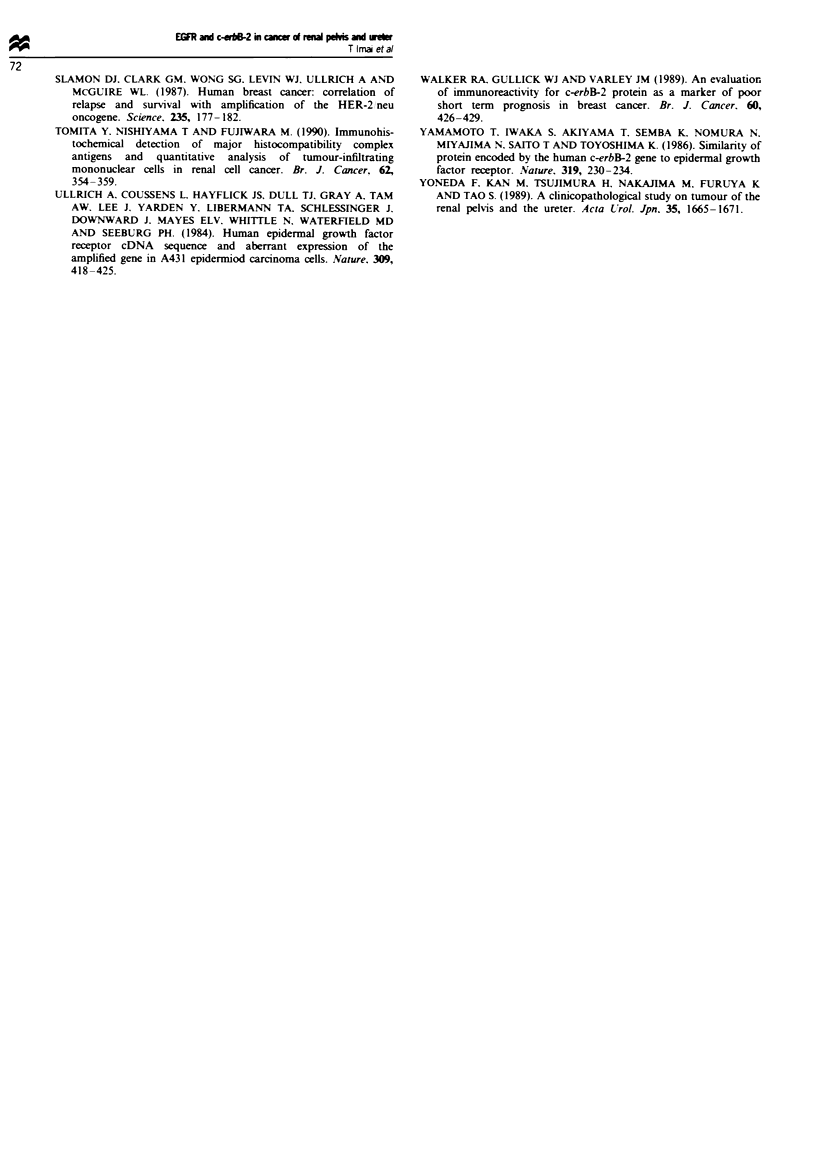# Significance of epidermal growth factor receptor and c-erbB-2 protein expression in transitional cell cancer of the upper urinary tract for tumour recurrence at the urinary bladder.

**DOI:** 10.1038/bjc.1995.14

**Published:** 1995-01

**Authors:** T. Imai, M. Kimura, M. Takeda, Y. Tomita

**Affiliations:** Department of Urology, Niigata University School of Medicine, Japan.

## Abstract

**Images:**


British Journal of Cancer (1995) 71, 69-72

© 1995 Stockton Press All rights reserved 0007-0920/95 $9.00

### Significance of epidermal growth factor receptor and c-*erb*B-2 protein expression in transitional cell cancer of the upper urinary tract for tumour recurrence at the urinary bladder

T Imai, M Kimura, M Takeda and Y Tomita

*Department of Urology, Niigata University School of Medicine, Asahimachi 1, Niigata 951, Japan.*

**Summary** An immunohistochemical study of the expression of epidermal growth factor receptor (EGFR) and c-*erb*B-2 protein was performed in fresh-frozen sections from 30 patients with transitional cell cancers (TCCs) of the upper urinary tract (15 renal pelvic cancers, 15 ureteral cancers) who underwent total nephroureterectomy. We followed them and examined whether TCC appeared in the urinary bladder. The follow-up period ranged from 116 to 2348 days (mean 666 days). The mean period until a secondary urinary bladder cancer appeared was 306 days (116-829 days). Thirteen of those 30 TCCs (43.3%) showed increased expression of EGFR, and 11 TCCs (36.7%) showed increased expression of c-*erb*B-2. In 12 of 30 patients (40.0%), a secondary urinary bladder cancer appeared after surgery. In only one of the ten patients (10.0%) whose tumours did not exhibit increased expression of either of these receptors the tumour recurred in bladder. On the other hand, in 11 of 20 (55.0%) patients whose tumours had increased EGFR and/or c-*erb*B-2 expression, secondary urinary bladder cancers recurred after surgery ($P<0.05$). Thus, the recurrence rate of TCCs with increased EGFR and/or c-*erb*B-2 expression was significantly higher than that of tumours showing no increased expression of these receptors ($P<0.01$). These results suggest that the immunohistochemical detection of the expression of EGFR and c-*erb*B-2 in urothelial cancers of the upper urinary tract might be a useful method for determining the likelihood of secondary bladder cancer recurrences.

**Keywords:** EGFR; c-*erb*B-2 protein; secondary urinary bladder cancer

Epidermal growth factor receptor (EGFR) is a transmembrane glycoprotein which has tyrosine kinase activity and augments cell proliferation on interaction with its ligand, EGF. EGFR is expressed at increased levels in certain carcinomas, such as breast cancer (Sainsbury *et al*., 1987; Harris *et al*., 1989), oesophageal cancer (Ozawa *et al*., 1989) and urinary bladder cancer (Neal *et al*., 1985, 1990; Lipponen and Eskelinen, 1994). In those carcinomas, the expression of the EGFR appears to be associated with the patient's prognosis. The c-*erb*B-2 gene encodes a transmembrane glycoprotein which is supposed to have similar function to EGFR (Yamamoto *et al*., 1986; Lee *et al*., 1989). Immunohistological studies have revealed that the c-*erb*B-2 protein is commonly located in fetal epithelial cells but is barely detectable in post-natal tissues (Mori *et al*., 1989). Therefore, this protein has been assumed to be a growth factor receptor that plays a role in mitogenic signalling in the fetal epithelium. The overexpression of c-*erb*B-2 has been shown to be correlated with lymph node metastasis and a poorer prognosis in those with breast cancer, suggesting that c-*erb*B-2 may play an important role in the progression of cancers (Slamon *et al*., 1987; Walker *et al*., 1989).

Transitional cell cancers (TCCs) in the urinary tract appear to occur multicentrically. After a total nephroureterectomy for TCCs of the renal pelvis or ureter, patients require continued surveillance, because the incidence of a subsequent bladder cancer is about 20-40% (Batara and Grabsteld, 1976; Kakizoe *et al*., 1980; Nielsen and Ostri, 1988; Catalona, 1992). Although several studies have been performed to determine the relationship between urothelial cancers of the upper urinary tract and secondary urinary bladder cancers after total nephroureterectomy, none of the criteria examined was found to be predictive of recurrence in urinary bladder. We reported that EGFR and c-*erb*B-2 were also overexpressed in TCCs in ureter or renal pelvis, and that the degree of expression was correlated with the histopathological grade of the tumour and the degree of invasion (Kimura *et al*., 1992). In this study, our interest was focused on correlation between the degree of expression of EGFR or c-*erb*B-2 and recurrence in the urinary bladder after surgical removal of TCCs in ureter or renal pelvis. Our data show that recurrence in the bladder occurs more frequently in patients with EGFR- and/or c-*erb*B-2-overexpressing TCCs of the upper urinary tract.

Correspondence: T Imai, Department of Urology, Niigata University School of Medicine, Asahimachi 1, Niigata 951, Japan
Received 23 February 1994; revised 12 July 1994; accepted 12 August 1994

### Materials and methods

### Patients

Thirty patients (24 males and six females; mean age 67.9 years, range 53-81) who had undergone total nephroureterectomy for renal pelvic (15 cases) or ureteral cancer (15 cases) were admitted to this study. All the tumours were diagnosed pathologically as TCC. The patients were not treated with either chemotherapy or radiotherapy before surgery. Patients with bladder cancer or distant metastasis before surgery or with lymph node metastasis identified by histopathology after surgery were excluded from this study. Seven patients had no invasion of the lamina propria (pTa), eight showed lamina propria invasion (pT1), seven had invasion of superficial muscle (pT2) and eight had invasion of the deep muscle (pT3). Pathological grade 1 tumours (high differentiation) occurred in five patients, grade 2 (moderate differentiation) in 20 and grade 3 (poor differentiation) in five.

### Immunoperoxidase staining

Frozen sections were cut at 5 μm, air dried for 30 min and fixed in cold acetone for 10 min. One section from each sample was subjected to immunohistochemical staining for EGFR and c-*erb*B-2 and to examination of histopathology. The immunohistochemical study was performed using the streptavidin-biotin bridge technique as described elsewhere (Tomita *et al*., 1990). Briefly, after rehydration with phosphate-buffered saline (PBS), the sections were incubated in 20% normal sheep serum or 20% normal donkey serum (Antibodies, Davis, CA, USA) in PBS for 30 min.

Endogenous biotin was blocked using the Endogenous Biotin Blocking Kit (Vector Laboratories, Burlingame, CA, USA). The sections then were incubated with mouse monoclonal anti-human EGFR antibodies (Transformation Research, Framingham, MA, USA) or rabbit polyclonal anti-human c-*erb*B-2 antibodies (Nichirei, Tokyo, Japan) for 60 min followed by incubation with biotinylated sheep anti-mouse or donkey anti-rabbit serum (Amersham International, Amersham, Bucks, UK) containing 20% human type AB serum (Biological Speciality, Lansdale, PA, USA). Subsequently, they were incubated for 45 min with streptavidin peroxidase (Amersham). Each step was followed by three washes in PBS. Finally, the sections were immersed in 0.05% diaminobenzidine (Sigma, St Louis, MO, USA) and 0.01% hydrogen peroxide in 0.05 M Tris-HCl buffer for 3-5 min to visualise the reaction products. After washing the sections in tap water, they were counterstained in Mayer's haematoxylin and mounted with Eukitt (O Kuldler, Freiburg, Germany) after dehydration in graded ethanol and xylene solutions. For determination of the optimal dilution of each antibody, A 431 cells (Ullrich *et al*., 1984) for EGFR expression and MKN7 cells (Yamamoto *et al*., 1986) for c-*erb*B-2 expression were examined. For the evaluation of staining, the approximate percentage of positive cells was estimated in randomly selected fields at a magnification of × 100 under a microscope equipped with a graticule. In ten examples of normal transitional epithelium (two from renal pelvis, three from ureter and five from urinary bladder) we could detect only less than 25% positively cells (Kimura *et al*., 1992). Therefore, when the percentage of positive TCCs exceeded 25%, the specimens were scored as exhibiting increased expression for each receptor protein.

### Results

Thirteen of the 30 TCCs (43.3%) exhibited increased expression of the EGFR, and 11 TCCs (36.7%) showed increased c-*erb*B-2 expression (Figure 1). Increased c-*erb*B-2 expression was not associated with histological grade or tumour stage, however increased EGFR expression was found more frequently in patients having invasion of the deeper layer of the renal pelvis or ureter ($P<0.05$, $\chi^2 = 4.887$, Yates' correction) (Table I). All patients with stage pT3 cancers showed overexpression of the EGFR or c-*erb*B-2 proteins.

Secondary urinary bladder cancers occurred in 12 of the 30 patients (40.0%) after total nephroureterectomy. Although higher grade and higher stage tumours tended to recur frequently in the bladder, this difference was not statistically significant in this study (Table II). Recurrence in the bladder occurred in only one of the ten patients (10.0%), whose tumour showed no increase in expression of either receptor. On the other hand, in 11 of 20 (55.0%) patients with tumours showing increased EGFR or c-*erb*B-2 expression, a secondary urinary bladder cancer occurred after surgery, and this difference was significant statistically ($P<0.05$, Fisher's exact probability method) (Table III). The follow-up period ranged from 116 to 2348 days (mean 666 days). The mean period until a secondary urinary bladder cancer occurred was 306 days (116-829 days). The recurrence rate of TCCs showing increased EGFR and/or c-*erb*B-2 expression was significantly higher than that of tumours showing no increased expression of these receptor proteins (Kaplan-Meier method, $P<0.01$, generalised Wilcoxon test) (Figure 2).

**Figure 1** Grade 1, pTa, transitional cell cancer of the ureter positively stained with both EGFR (**a**) and c-*erb*B-2 (**b**). Secondary urinary bladder cancer occurred in the patient after total nephroureterectomy. The interval until recurrence was 829 days. Bar = 75 μm.

**Table I** Expression of epidermal growth factor receptor (EGFR) and c-*erb*B-2 protein in relation to tumour grade and stage

| | *Grade* | | | *pT stage* | | |
|---|---|---|---|---|---|---|
| | *1* | *2* | *3* | ≤1 | ≥2 | |
| EGFR (%) | | | | | | |
| ≥25 | 3 | 5 | 5 | 3 | 10 | * |
| <25 | 2 | 15 | 0 | 12 | 5 | |
| c-*erb*B-2 (%) | | | | | | |
| ≥25 | 3 | 7 | 1 | 7 | 4 | |
| <25 | 2 | 13 | 4 | 8 | 11 | |

EGFR, epidermal growth factor receptor. $^{*}P<0.05$ ($\chi^2 = 4.887$, Yates' correction).

**Table II** Relationship between secondary bladder recurrence (12 of 30 patients) and tumour grade and stage

| | *Grade* | | | *pT stage* | | | |
|---|---|---|---|---|---|---|---|
| | *1* | *2* | *3* | *a* | *1* | *2* | *3* |
| Bladder recurrence | | | | | | | |
| (+) | 2 | 7 | 3 | 2 | 1 | 4 | 5 |
| (−) | 3 | 13 | 2 | 5 | 7 | 3 | 3 |

### Discussion

For patients suffering from renal pelvic cancer or ureteral cancer, continued follow-up is required because of the possibility of recurrence in the urinary bladder after total nephroureterectomy. In several reports, the histopathological grade of TCCs in the upper urinary tract was considered a factor predictive of such recurrence (Murphy *et al*., 1981; Krogh *et al*., 1991), but data from other series of patients, suggest that there is no relation between the recurrence rate and grade or stage (Yoneda *et al*., 1989). It seems that the classification of TCCs in the upper urinary tract by conventional pathological criteria does not always help in the prediction of bladder recurrence. Therefore, there is a need to

**Table III** Tumours grouped according to increased expression of EGFR and or c-*erb*B-2 *vs* no increased expression of either in relation to secondary bladder recurrence

| *EGFR* | *c-erbB-2* | *Bladder recurrence* (+) | (−) | |
|---|---|---|---|---|
| ⩽25% and or | ⩾25% | 11 | 9 | * |
| <25% and | <25% | 1 | 9 | |

EGFR, epidermal growth factor receptor. *$P<0.05$ (Fisher's exact probability method).

**Figure 2** Rate of secondary bladder recurrence: tumour with increased expression of EGFR and or c-*erb*B-2 (solid line) and tumours with no increased expression of either (dotted line) (Kaplan–Meier method, $P<0.01$, generalised Wilcoxon test).

identify a characteristic of tumour cells associated with the risk of recurrence in the bladder to enable identification of patients with either a low recurrence rate (who do not need follow-up) or a high recurrence rate (who might benefit from more intensive treatment and frequent examination).

Clinical and urinary tract mapping studies suggest that TCC is usually a field change disease with tumours arising at different times and sites in the urothelium. This suggests the possibility of a polyclonal aetiology of urothelial cancer. However, it does not exclude the possibility that, in some cases, multiple tumours are derived from a single-cell clone that has disseminated to other sites in the urinary tract by implantation. The determination of quantitative or qualitative changes in the expression of oncogenes and their protein products might be useful for classifying tumours into different prognostic categories, since such changes at the cellular level may lead directly to alterations in tumour behaviour. Such information might well supplement traditional prognostic factors such as tumour grade and stage. Increased expression of the epidermal growth factor receptor (EGFR) and c-*erb*B-2 protein is found in urothelial cancers, and has been reported to be associated with tumour invasiveness, pathological malignancy, distant metastasis and clinical prognosis (Neal *et al.*, 1985, 1990; Moriyama *et al.*, 1991; Kimura *et al.*, 1992; Lipponen and Eskelinen, 1994). In this study, secondary urinary bladder cancers occurred in 12 of 30 patients with renal pelvic TCC or ureteral TCC after total nephroureterectomy. Eleven of the 20 patients (55.0%) showing increased expression of EGFR and/or c-*erb*B-2 developed a recurrence in the urinary bladder. In contrast only one of the ten patients (10.0%) whose TCCs overexpressed neither EGFR nor c-*erb*B-2 exhibited a recurrence ($P<0.05$). On the other hand, we could not find any significant correlation between tumour grade or stage and incidence of urinary bladder recurrence of tumour. Furthermore, the mean period until recurrence in the urinary bladder was 306 days, and there was a significant difference in the recurrence rate in TCCs with increased EGFR or c-*erb*B-2 expression and the recurrence rate of TCCs with no increased expression ($P<0.01$). These results suggest that the immunohistochemical detection of EGFR and c-*erb*B-2 in urothelial cancers of the upper urinary tract might be a useful method for determining the recurrence potential in the urinary bladder.

**Acknowledgements**
The authors thank Professor S Sato, Dr T Tanikawa, Dr A Katagiri and Dr S Wakatsuki for their assistance and useful advice.

**References**

BATARA M AND GRABSTELD H. (1976). Upper urinary tract urothelial tumours. *Urol. Clin. N. Am.*, **3**, 79–86.
CATALONA WJ. (1992). Urothelial tumours of the urinary tract. In *Campbell's Urology*, Walsh PC *et al.* (eds) pp. 1094–1158. WB Saunders: Philadelphia.
HARRIS AL, NICHOLSON S, SAINSBURY JRC, FARNDON J AND WRIGHT C. (1989). Epidermal growth factor receptors in breast cancer: association with early relapse and death, poor response to hormones and interactions with neu. *J. Steriod Biochem.*, **34**, 123–131.
KAKIZOE T, FUJITA J, MURASE T, MATSUMOTO K AND KISHI K. (1980). Transitional cell carcinoma of the bladder in patients with renal pelvic and ureteral cancer. *J. Urol.*, **124**, 17–19.
KIMURA M, TOMITA Y, NISHIYAMA T, SATO S AND OHMORI K. (1992). Expression of epidermal growth factor receptor and c-*erb*B-2 product in transitional cell carcinoma. *J. Jpn Soc. Cancer Ther.*, **27**, 1132–1138.
KROGH J, KVIST E AND RYA B. (1991). Transitional cell carcinoma of the upper urinary tract – prognostic variables and postoperative recurrence. *Br. J. Cancer*, **67**, 32–36.
LEE J, DULL TJ, LAX I, SCHLESSINGER J AND ULLRICH A. (1989). HER2 cytoplasmic domain generates normal mitogenic and transforming signals in a chimeric receptor. *EMBO J.*, **8**, 167–173.
LIPPONEN P AND ESKELINEN M. (1994). Expression of epidermal growth factor receptor in bladder cancer as related to established prognostic factors, oncoprotein (c-*erb*B-2, p53) expression and long-term prognosis. *Br. J. Cancer*, **69**, 1120–1125.
MORI S, AKIYAMA T, YAMADA Y, MORISHITA Y, SUGAWARA I, TOYOSHIMA K AND YAMAMOTO T. (1989). c-*erb*B-2 gene product: a membrane protein commonly expressed on human fetal epithelial cells. *Lab. Invest.*, **61**, 93–97.
MORIYAMA M, AKIYAMA T, YAMAMOTO T, KAWAMOTO T, KATO T, SATO K, WATANUKI T, HIKAGE T, KATSUTA N AND MORI S. (1991). Expression of the c-*erb*B-2 gene product in urinary bladder cancer. *J. Urol.*, **145**, 423–427.
MURPHY DM, ZINKE H AND FURLOW WL. (1981). Management of high grade transitional cell cancer of the upper urinary tract. *J. Urol.*, **125**, 25–29.
NEAL DE, MARSH C, BENETT MK, ABEL PD, HALL RR, SAINSBURY JRC AND HARRIS AL. (1985). Epidermal growth factor receptors in human bladder cancer: comparison of invasive and superficial tumours. *Lancet*, **i**, 366–368.
NEAL DE, SHARPLES L, SMITH K, FENNELLY J, HALL RR AND HARRIS AL. (1990). The epidermal growth factor and the prognosis of bladder cancer. *Cancer*, **65**, 1619–1625.
NIELSEN K AND OSTRI P. (1988). Primary tumours of the renal pelvis: evaluation of clinical and pathological features in a consecutive series of 10 years. *J. Urol.*, **140**, 19–21.
OZAWA S, UEDA M, ANDO N, SHIMIZU N AND ABE O. (1989). Prognostic significance of epidermal growth factor receptor in esophageal squamous cell carcinomas. *Cancer*, **63**, 2169–2173.
SAINSBURY JRC, FARNDON JR, NEELHAM GK, MALCOLM AJ AND HARRIS AL. (1987). Epidermal-growth-factor receptor status as predictor of early recurrence of and death from breast cancer. *Lancet*, **i**, 1398–1402.

SLAMON DJ, CLARK GM, WONG SG, LEVIN WJ, ULLRICH A AND McGUIRE WL. (1987). Human breast cancer: correlation of relapse and survival with amplification of the HER-2/neu oncogene. *Science*, **235**, 177–182.

TOMITA Y, NISHIYAMA T AND FUJIWARA M. (1990). Immunohistochemical detection of major histocompatibility complex antigens and quantitative analysis of tumour-infiltrating mononuclear cells in renal cell cancer. *Br. J. Cancer*, **62**, 354–359.

ULLRICH A, COUSSENS L, HAYFLICK JS, DULL TJ, GRAY A, TAM AW, LEE J, YARDEN Y, LIBERMANN TA, SCHLESSINGER J, DOWNWARD J, MAYES ELV, WHITTLE N, WATERFIELD MD AND SEEBURG PH. (1984). Human epidermal growth factor receptor cDNA sequence and aberrant expression of the amplified gene in A431 epidermiod carcinoma cells. *Nature*, **309**, 418–425.

WALKER RA, GULLICK WJ AND VARLEY JM (1989). An evaluation of immunoreactivity for c-*erb*B-2 protein as a marker of poor short term prognosis in breast cancer. *Br. J. Cancer*, **60**, 426–429.

YAMAMOTO T, IWAKA S, AKIYAMA T, SEMBA K, NOMURA N, MIYAJIMA N, SAITO T AND TOYOSHIMA K. (1986). Similarity of protein encoded by the human c-*erb*B-2 gene to epidermal growth factor receptor. *Nature*, **319**, 230–234.

YONEDA F, KAN M, TSUJIMURA H, NAKAJIMA M, FURUYA K AND TAO S. (1989). A clinicopathological study on tumour of the renal pelvis and the ureter. *Acta Urol. Jpn*, **35**, 1665–1671.